# Molecular Dynamics Simulation of the Viscosity Enhancement Mechanism of P-n Series Vinyl Acetate Polymer–CO_2_

**DOI:** 10.3390/polym16213034

**Published:** 2024-10-29

**Authors:** Hong Fu, Yiqi Pan, Hanxuan Song, Changtong Xing, Runfei Bao, Kaoping Song, Xindong Fu

**Affiliations:** 1 Unconventional Oil and Gas Research Institute, China University of Petroleum, Beijing 102249, China; 2 Research Institute of Exploration and Development of Daqing Oilfield Company Ltd., Daqing 138000, China; 3 Daqing Drilling Engineering Company Underground Operation Company Technology Research Institute, Songyuan 138000, China

**Keywords:** molecular dynamics simulation, polymers, CO_2_ binder, accessible surface area

## Abstract

Carbon dioxide (CO_2_) drive is one of the effective methods to develop old oil fields with high water content for tertiary oil recovery and to improve the recovery rate. However, due to the low viscosity of pure CO_2_, it is not conducive to expanding the wave volume of the mixed phase, which leads to difficulty utilizing the residual oil in vertical distribution and a low degree of recovery in the reservoir. By introducing viscosity enhancers, it is possible to reduce the two-phase fluidity ratio, expanding the degree of longitudinal rippling and oil recovery efficiency. It has been proven that the acetate scCO_2_ tackifier PVE can effectively tackify CO_2_ systems. However, little research has been reported on the microscopic viscosity enhancement mechanism of scCO_2_ viscosity enhancers. To investigate the influence of a vinyl acetate (VAc) functional unit on the viscosity enhancement effect of the CO_2_ system, PVE (Polymer–Viscosity–Enhance, P-3) was used as the parent, the proportion of VAc was changed, and the molecules P-1 and P-2 were designed to establish a molecular dynamics simulation model for the P-n-CO_2_ system. The molecules in the system under the conditions of 70 °C-10 MPa, 80 °C-10 MPa, and 70 °C-20 MPa were simulated; the viscosity of the system was calculated; and the error between the theoretical and simulated values of the viscosity in the CO_2_ system was relatively small. The difference between P-n molecular structure and system viscosity was analyzed at multiple scales through polymer molecular dynamics simulations and used the molecular radial distribution function, system density, accessible surface area, radius of gyration, minimum intermolecular distance, and minimum number of intermolecular contacts as indicators. This study aimed to elucidate the viscosity enhancement mechanism, and the results showed that the higher the proportion of VAc introduced into the molecules of P-n-scCO_2_ viscosities, the larger the molecular amplitude, the larger the effective contact area, and the greater the viscosity of the system. Improvement in the contact efficiency between the ester group on the P-n molecule and CO_2_ promotes the onset of solvation behavior. This study on the microscopic mechanism of scCO_2_ tackifiers provides a theoretical approach for the design of new CO_2_ tackifiers.

## 1. Introduction

Supercritical carbon dioxide (scCO_2_) fluids as an enhanced recovery (EOR) technology can improve crude oil recovery in low permeability reservoirs through mechanisms such as reducing crude oil viscosity and lowering interfacial tension [1,2]. The current conventional CO_2_ oil drive system has low viscosity, which makes it difficult to overcome the problem of fluidity ratio, and the viscosity refers to the trigger of large-scale gas flushing, which adversely affects normal production [3,4]. In comparison, it is more economical to optimize the performance of CO_2_ directly—the introduction of viscosity enhancers to regulate the fluidity ratio is one of the effective measures to solve the problem [5].

Current research on scCO_2_ tackifiers is mainly divided into two categories: physical and chemical tackifiers [6,7]: Chemical viscosity enhancement is achieved by binding the transport rate of CO_2_ molecules to achieve viscosity enhancement through intermolecular interactions, such as π–π stacking, to form a stable network structure. Physical viscosity, on the other hand, is mainly the use of intermolecular forces to reduce the fluid flow rate, in which case the apparent viscosity builder should have a better affinity for CO_2_. It can effectively improve the viscosity of CO_2_ [8,9,10]. According to current reports, the viscosity-increasing effect of chemical viscosity enhancers is much higher than that of physical viscosity enhancers, but most of the agents have certain limitations in terms of synthesis cost, solubility, effective concentration, stability, viscosity retention rate, etc. [11,12]. Currently, viscosity enhancers are mainly used in fracking, but are not suitable for direct application in oil production due to the significant difference in effective concentration.

Although supercritical carbon dioxide thickeners have been designed for more than 50 years, there are currently no widely used and effective scCO_2_ viscosity enhancers in the field of oil repelling. In addition, the research on existing CO_2_ viscosity enhancers mainly focuses on the macroscopic oil-repellent effect and the contact effect of surface activators or foam viscosity enhancers at the oil–water interface, while research indicates that the microscopic viscosity enhancement mechanism has not been perfected yet.

Based on the problems of existing viscosity enhancers, good CO_2_ viscosity enhancers for oil recovery should be designed to minimize the proportion of chemical elements that are not present in the original reservoir, and rely as much as possible only on C, H, and O to form the functional groups of the molecule [13,14]. In previous work, authors have pre-modified polyvinyl acetate ester (PVE), which has good CO_2_ affinity but poor solubility [15]. To improve the thermal stability of the polymer, an acetate-based viscosity builder, PVE, was designed. It has been proven that PVE can realize effective viscosity increase for the CO_2_ system, inhibit gas flushing, and realize effective oil recovery increase [16], which solves the contradiction between low reagent concentration and appropriate viscosity increase in production [17,18]. It was concluded that C=O in the system has a strong affinity for CO_2_, which can effectively limit the rate of movement of CO_2_ and effectively enhance the viscosity of the system. Through experiments to adjust the system temperature and pressure, it is clear that PVE can effectively inhibit gas flushing, but the viscosity enhancement micro-mechanism has not been explored in depth.

Computational chemistry methods can elucidate intermolecular interaction mechanisms at the microscopic level. Researchers apply it extensively at the molecular level to reveal microscopic interactions between CO_2_ and polymer molecules. R FARZIN and J INSEOK investigated the effect of temperature changes on the surface decomposition of polymers in conjunction with molecular dynamics simulations [19,20]; Guan and his colleagues found that sulfonation-modified PCE had superior dispersion stability, but did not go on to investigate the adsorption behavior of the modification in depth [21]; Gurina et al. [22] suggested that scCO_2_ significantly promotes the swelling behavior of PMMA. At present, in the field of oilfield development, some experts have studied the interfacial wetting relationship between oil droplets and pore throats through molecular dynamics theory; analyzed the effect of temperature, crude oil components, and other factors on the minimum mixing phase pressure [15]; and explored the relationship between the system viscosity and density changes in the oil-repellent system on breakthrough time [23]. However, few studies have been reported on the microscopic mechanism of the interaction between CO_2_ and tackifiers. This paper combines molecular simulation software to construct a P-n series polymer–CO_2_ mixing model to calculate the viscosity variation rule of the system after equilibrium. The distribution of CO_2_ molecules with P-n molecules in the post-equilibrium system was investigated by visualization of the model. Then, a multi-scale study was conducted to investigate the degree of structural changes within the P-n molecule before and after equilibrium, and the law of influence of changes in the molecular weight of P-n and the total number of molecules in the model on the structure of the P-n molecule. By comparing the range of fluctuation of each atom in the molecular chain, the flexibility of each functional group in the molecular chain segment is investigated, and the minimum distance between molecules after model equilibrium is calculated. The variation rules of viscosity and contact area of the P-n-scCO_2_ system under different concentrations, temperatures, and pressures is elucidated, and the trend of CO_2_ changes around P-n molecules at multiple scales (molecules, groups, and atoms) is analyzed. This paper provides directions for studying the viscosity enhancement mechanism of acetate polymer-based CO_2_ viscosity enhancers.

## 2. Materials and Methods

**Figure 1:**
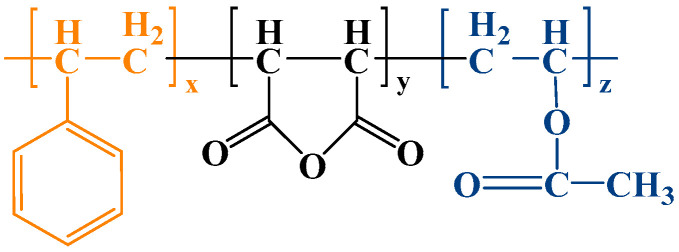
Structure of ideal viscosifier.

Considering the polymer has polydispersity, the groups contained in the three designed polymers are nonpolar or weakly polar. To guarantee the lowest energy of the system and simplify the discussion, according to previous experience, three kinds of P-n molecules are set up as a linear symmetric structure, i.e., St is located at both ends of the molecule, linking the MA, and the VAc is in the center. The structure of the polymers is shown in Figure 1.

Previous experimental studies have shown that when the content of VAc is too high, the electron-supplying group (-OCOCH_3_) in the structure of the VAc is exceptionally active, allowing for easy chain transfer and chain termination reaction [24], and the polymerization system is prone to exothermic self-polymerization of the VAc, which makes it difficult to maintain the temperature of the system at a constant level, which in turn leads to over-polymerization. Therefore, the percentage of VAc should be controlled at less than 35%. When the ratio of MA to VAc exceeds 4:2.5, it is easier to generate an MA-VAc dimer, and the trimer yield after purification is obviously low; St is more stable in free radical polymerization, and the best yield is achieved when the molar ratio is about 1/4 of MA. Based on the above reasons, the final molecularly designed polymer monomers were at a ratio of 1:4:1 to 1:4:2.5. The molecular structures were plotted based on the substance-to-quantity ratios of the three polymerization products, and the atomic compositions of the three polymers are shown in Table 1.

### 2.1. Single-Molecule Modeling and Structure Optimization

The ball-and-stick structures of the three P-n molecules and the CO_2_ molecule were plotted separately using molecular dynamics simulation visualization software VMD win64-1.9.3 (Visual Molecular Dynamics win64-1.9.3) based on the component molar ratios in Table 2. To distinguish the different P-n molecular chain arrangements more clearly, the initial structures of the three polymers were set to be linearly symmetric, and CO_2_, which does not have a dipole moment, was also linear. To reduce the influence of irrational configurations on the intermolecular forces and to describe more accurately the bond energies and bond angles of the viscosity builder macromolecules, three molecules were subjected to geometric optimization (Geometry Optimization) in combination with the quantum chemical program Orca5.0 [25,26] according to the theory of B3LYP/6-31G(d,p), before the introduction of the monomeric molecules into the system. The analysis process was performed without solvent by default, and the comparison of optimized main chain-functional group dihedral angles before and after optimization is shown in the Table 2 below.

**Figure 2:**
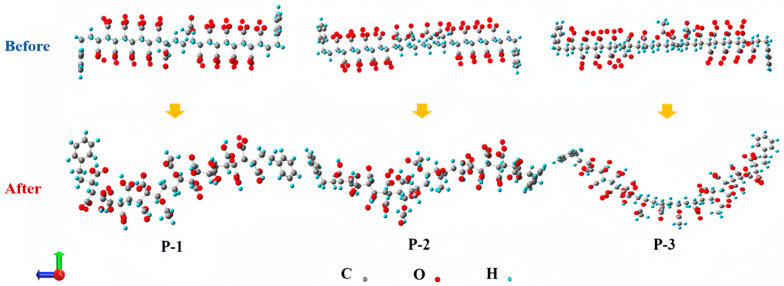
Conformation of three different polymer structures before and after optimization.

Energy reduction and convergence after optimization of the dihedral angle of the main chain-functional functional group: After comparison, it can be seen that the maximum force, square root force, maximum displacement, and square mean root displacement of the optimized P-n molecular structures are less than 0.00045, 0.00030, 0.00180, and 0.00120, respectively, indicating that the three optimized P-n molecular structures correspond to the energy minima on the potential energy surface. The conformational optimization provides a more reasonable molecular structure for the later introduction of carbon dioxide hybrid system calculation. After measurement, the optimized final dimensions of the three P-n molecules were obtained as 31.55 Å × 14.24 Å × 8.12 Å, 35.49 Å × 14.449 Å × 11.59 Å, and 35.77 Å × 19.86 Å × 9.74 Å, and the optimized molecular structures are shown in Figure 2. The different atoms are color-coded for ease of differentiation: C atoms are gray ●, O atoms are red ●, and H atoms are blue ●. According to the optimization results, the final equilibrium structures of the three P-n molecules are still linear, but none of them are ideal linear structures.

### 2.2. Modeling and Simulation Method for P-n-scCO_2_ System

Molecular dynamics simulation (MD) of the CO_2_-adhesive system was carried out using Gromacs 2020.6 software and the charmm36 force field, and the system energy was calculated as shown in Equation (1). MD simulations were performed under constant pressure, constant temperature (298.15 K, 1 bar), and three-dimensional periodic boundary conditions (PBC). It is known that the test concentration of the system for indoor experiments is 0.2%, and if the idealized model is directly based on the actual number of polymers, it is likely that a large portion of the CO_2_ in the system will not be able to achieve effective contact with the polymers. Therefore, to better represent the polymer–polymer and polymer–CO_2_ molecule interactions, the number of polymers in the system was scaled up, which only affects the multiplicity of the viscosity increase, and does not significantly affect the viscosity enhancement pattern or any other analytical-related data. Eventually, in the MD simulation, the composition inside the design box was 50 polymers + 4000 CO_2_ and 100 polymers + 4000 CO_2_, and the blank control was 4000 CO_2_. After confirming that the setup of the base file was complete, several P-n molecules were inserted into the box with a length × width × height of 10 × 10 × 20 nm^3^ along with CO_2_ using the initial structure construction program, packmol. All molecules were put into the box at once during the insertion process to ensure that the molecules within the system were uniformly distributed within the box in the ideal state. The RESP method was used to calculate the molecular charge, and this part of the calculation was performed in the Multiwn 3.8 program.

(1) $$\begin{array}{c} E=\sum_{bonds}K_{b}{\left(l-l_{0}\right)}^{2}+\sum_{angle}K_{\theta }{\left(\theta -\theta_{0}\right)}^{2}+\sum_{UB}K_{UB}{\left(S-S_{0}\right)}^{2}\\ +\sum_{dihedral}V_{\omega }\left[1+\cos\left(n\omega -\omega_{0}\right)\right]+\sum_{out-of-plane}K_{\chi }{\left(\chi -\chi_{0}\right)}^{2}\\ +\sum_{nonbond}\varepsilon_{ij}\left[{\left(\frac{R_{ij}}{r_{ij}}\right)}^{12}-2{\left(\frac{R_{ij}}{r_{ij}}\right)}^{6}\right]+\sum_{ele}\frac{q_{i}q_{j}}{4\pi \varepsilon r_{ij}} \end{array}$$

where $\sum_{bonds}K_{b}{\left(l-l_{0}\right)}^{2}$, $\sum_{angle}K_{\theta }{\left(\theta -\theta_{0}\right)}^{2}$, $\sum_{UB}K_{UB}{\left(S-S_{0}\right)}^{2}$, $\sum_{dihedral}V_{\omega }\left[1+\cos\left(n\omega -\omega_{0}\right)\right]$, $\sum_{out-of-plane}K_{\chi }{\left(\chi -\chi_{0}\right)}^{2}$, $\sum_{nonbond}\varepsilon_{ij}\left[{\left(\frac{R_{ij}}{r_{ij}}\right)}^{12}-2{\left(\frac{R_{ij}}{r_{ij}}\right)}^{6}\right]$ and $\sum_{ele}\frac{q_{i}q_{j}}{4\pi \varepsilon r_{ij}}$ are the bond lengths, bond angles, dihedral angles, anomalous dihedral angles, van der Waals interactions, and potential energy for electrostatic interactions, respectively.

Pre-equilibrium energy minimization operations are first performed during molecular dynamics simulations: The purpose of the energy minimization operation on the system box is to ensure the most stable environment for the state of the microscopic system, which is mainly determined by which of the potential and non-potential energy derivatives is 0 [27]. Depending on the size of the polymer–CO_2_ system and the types of molecules in the box, the computational convergence efficiency is low if the Steepest Descent (SD) method is used for the first level of derivation. If the Newton–Raphson method (Newton’s method) is used for the second level derivation, although there is some improvement in accuracy, Newton’s method requires the calculation of a large number of Hess matrices, which is computationally expensive, and both of the above methods do not apply to the calculations in this section. Therefore, in this section, the Conjugate Gradient (CG) method, which is better optimized and more computationally efficient among the current large-scale nonlinear algorithms, is used for the calculation of energy minimization [28]. The force field equations involved in the calculation are as follows:

(2) $$\vec{v_{k}}=-\vec{g_{k}}+\gamma_{k}\vec{v_{k-1}}$$

(3) $$\gamma_{k}=\frac{\vec{g_{k}}\times \vec{g_{k}}}{\vec{g_{k-1}}\times \vec{g_{k-1}}}$$

where $v_{k}$, $\gamma_{k}$, and $\vec{g_{k}}$ are the search direction at step *k*, the linear composition coefficients, and the negative gradient direction at step *k*, respectively.

After energy minimization, the system was sequentially subjected to normal–variable system equilibrium (NVT) and isothermal–isobaric system equilibrium (NPT), where V-rescale was used for the NVT system and constant pressure was used for the NPT system to control the temperature and pressure using the Parrinello–Rahman and Nose–Hoover pressure [29,30,31]. Considering the critical temperature and pressure of scCO_2_ (31.26 °C, 7.38 MPa). Calculations were carried out at 70 °C-10 MPa, 80 °C-10 MPa, and 70 °C-20 MPa, respectively, with a step size of 1 fs, in which the NVT was run for 5 ns to achieve pressure stabilization, and then the structure of the last frame of the NVT was succeeded by the NPT for 30 ns by the same step size, after which the entire box of molecules tended to be tightly stacked and there was no cavity, indicating that the system had reached equilibrium.

The analyses of the microscopic mechanisms in this section are all based on the above equilibrium structure, and the last 5 ns trajectory after the equilibrium of the molecular dynamics simulation is intercepted for the discussion of the minimum intermolecular distance and the number of molecular contact groups. To improve the accuracy of the calculations and to guarantee that the calculations cover the forces and velocities of each atom, the calculations related to energy, radial distribution, density, etc., in this chapter are performed using .trr binary trajectory files instead of .xtc analog trajectory single-precision files.

## 3. MD Calculated Equilibrium Determination Method

The energy of the whole system is first calculated, and this is used as a criterion for determining that the whole system has reached equilibrium. The energy calculation formula is shown in Equation (4).

(4) $$U=\langle E\rangle =\frac{1}{M}\sum_{i=1}^{M}E_{i}$$

**Figure 3:**
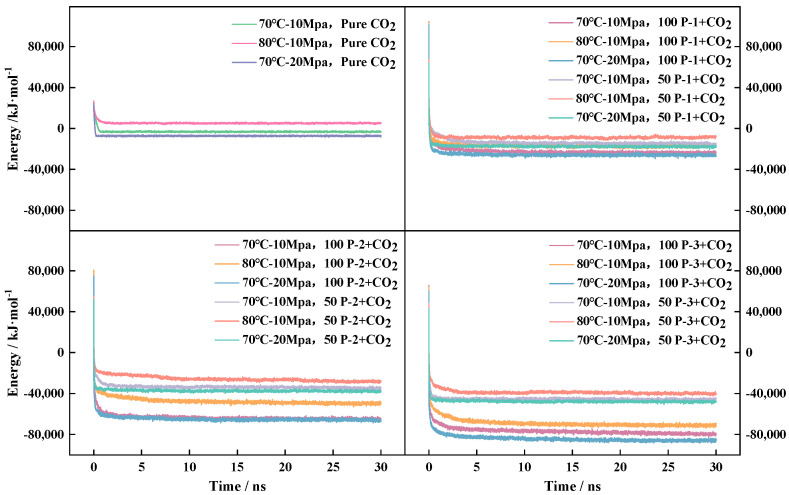
Schematic diagram of equilibrium energy fluctuation of thickener systems with different concentrations.

The calculated energy fluctuations in the system are shown in Figure 3. According to the law of motion of the energy curve, during the MD simulation, in the first 2 ns (2000 ps), the energy of the system decreases rapidly, and the energy of each system tends to equilibrate when it reaches 2 ns. The energy fluctuation of the system is stable between 2 ns and 30 ns, and the system can be considered to have achieved equilibrium.

A comparison of the energy calculation data shows that a single system (pure CO_2_) has the lowest energy loss when moving to equilibrium. According to the law of intermolecular action, polar nonpolar van der Waals forces change, and the energy required to overcome the motion between different molecules varies by polarity. Comparing the introduction of the same polymer system, under the same ambient temperature–pressure conditions, the higher the number of polymers introduced into the system, the higher the energy loss to achieve an equilibrium system. Due to the different number of polymers introduced into the system, the system converges to different energy values, respectively, and the greater the number of P-n molecules, the greater the energy loss for eventual convergence to equilibrium.

In addition to the influence of the molecular composition of the system and the total number of molecules in the system, the environment of the system also has some influence on the energy balance. According to the convergence trend of energy curves between different systems, in the same system with 70 °C-10 MPa compared to 70 °C-20 MPa system energy convergence is faster, energy reduction range is smaller, 80 °C-10 MPa is slower, the energy reduction range is larger, and the lower the temperature, the easier it is for the pressure system to converge to the equilibrium state. In both CO_2_ and polymer-CO_2_ systems, increasing the temperature promotes molecular thermal motion and reduces the energy loss for the system to achieve equilibrium. However, too high a temperature is rather detrimental to the equilibrium of the system, whereas the pressure is just the opposite [32], and elevating the pressure will inhibit the molecular thermal motion and favor the achievement of equilibrium. Based on the above analysis, the effect of pressure on the system energy is significantly higher than the effect of temperature on the system energy in the three polymer–CO_2_ systems.

## 4. Analysis of MD Calculations

The research objective of this chapter is to explore the viscosity enhancement mechanism of the polymer–CO_2_ system based on MD calculations; therefore, firstly, we verify the consistency of the viscosity change law of the system under different concentration and environmental (temperature and pressure) conditions in the equilibrium structure [33].

### 4.1. System Viscosity Study

There are two common methods of calculating MD viscosity, both of which require a continuation run of the resultant file after realizing NPT equilibrium, one of which is to obtain the viscosity inverse 1/viscosity via the Energy command and then convert it. The other method uses the periodic perturbation method for direct system viscosity calculation, and both methods are combined with the Green–Kubo viscosity equation [34]:

(5) $$\eta =\frac{V}{k_{B}T}{\int_{0}^{\infty }\langle P_{xz}\left(t_{0}\right)P_{xz}\left(t_{0}+t\right)\rangle }_{t_{0}}dt$$

(6) $$\eta =\underset{t\to \infty }{\lim}\frac{1}{2}\frac{V}{k_{B}T}\frac{d}{dt}\langle {\left(\int_{t_{0}}^{t_{0}+t}P_{xz}\left(t^{\prime }\right)dt\right)}^{2}\rangle$$

where *k*_B_, *V*, and *T* are the Boltzmann constant, temperature, and volume of the simulation box, respectively.

**Figure 4:**
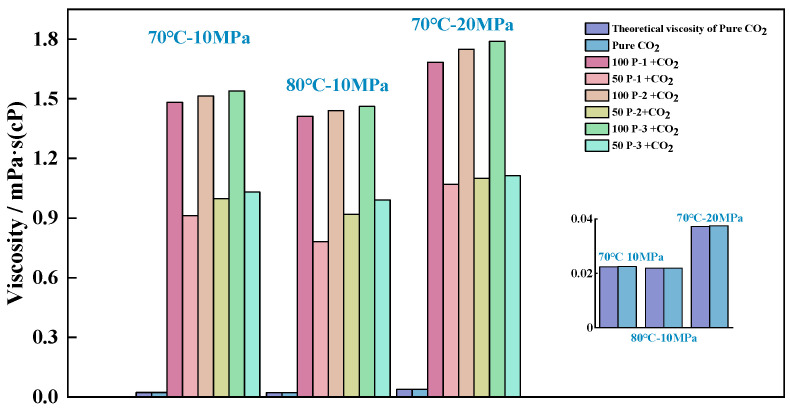
Comparison of viscosity of MD-calculated systems under different environmental conditions.

To compare the reliability of the two calculation methods in the molecular simulation model in this section, a blank control group—pure CO_2_—was used to evaluate the accuracy of the viscosity calculation results under the same conditions: the pure CO_2_ viscosities at 70 °C and 10 MPa were calculated to be 0.02235 mPa-s and 0.02250 mPa-s, respectively, which compared with the theoretical standard CO_2_ viscosity of 0.0235 mPa-s, with error rates of 4.89% and 4.25%, respectively. The inverse method of viscosity calculation is lengthy due to the total number of available molecules. To achieve the calculation of equilibrium, at least 10 ns or more is needed to continue the calculation, while the second method of calculation’s error is much smaller, and the method dramatically reduces the length of the calculation; it only needs to be extended by 2 ps, and the results of the calculation are more accurate. Therefore, the viscosity data of the system in this subsection are obtained by the second method of calculation; the specific calculation results are shown in Figure 4.

Figure 4 shows the viscosity of different systems: the bottom right side depicts an enlargement of the comparison of the theoretical and calculated values of CO_2_ viscosity, in which it can be seen that there is a small difference between the theoretical and simulated calculated values. The reasons for this bias in MD calculations can be attributed to the different objects of calculation: While the viscosity is calculated experimentally as a force profile induced by an external force at the macroscopic level, the linear velocity profile of the system at equilibrium is studied in the simulations. The calculated computational–experimental error rate for all three sets of CO_2_ viscosity data is less than 0.5%, indicating that the bias will not affect the subsequent analysis of the objective law of viscosity change.

**Figure 5:**
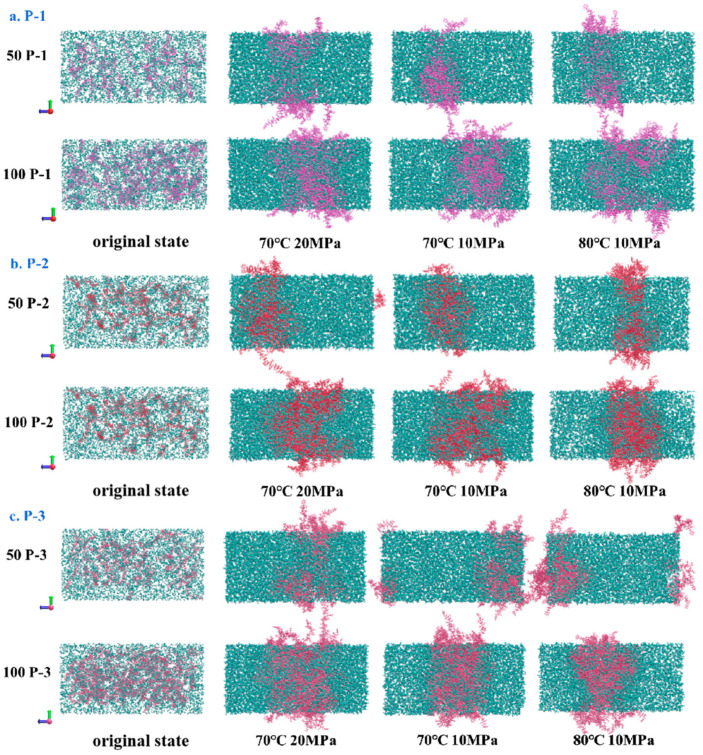
Molecular distribution before and after equilibrium of polymer–CO_2_ system.

By analyzing the various sets of data in Figure 5, it can be determined that when an acetate viscosity builder is introduced into CO_2_, the system viscosity is directly proportional to the number of molecules of the viscosity builder and the pressure and inversely proportional to the temperature, and the sensitivity of the system viscosity to the pressure is significantly higher than the temperature. The viscosities of the polymer–CO_2_ systems were ranked as Ƞ_100-P-3_ > Ƞ_100-P-2_ > Ƞ_100-P-1_, Ƞ_50-P-3_ > Ƞ_50-P-2_ > Ƞ_50-P-1_; compared with the CO_2_ in equilibrium state under the same environmental conditions, all the above systems realized effective viscosity improvement. MD calculations showed that the increase in viscosity increase was not mathematically exponential when comparing the introduction of 50 polymers and 100 polymers into the system, even though the viscosity of the system increased dramatically. For example, the introduction of 50 molecules of viscosity builder P-1 into the system allows for it to realize a 39.53% viscosity increase, while in the introduction of 100 molecules of P-1, viscosity increase is only 37.90% higher than that of the system with 50 P-1. To further examine the reasons for the emergence of the above patterns at the microscopic level, the distribution of CO_2_ around each viscosity builder molecule and the intermolecular interaction relationships were further investigated.

### 4.2. Visualization of Molecular Distribution Patterns

After confirming the effective viscosity increase in the system, to further study the polymer distribution law in the system under the equilibrium conditions, VMD was used to visualize and analyze the distribution of various types of molecules in the equilibrium calculation result file. Since each component within the system contains a large number of C and O elements, to facilitate the differentiation, the system gro file was categorized and colored according to the molecular nomenclature in the interface, see Figure 5. Here, the P-1 molecule is labeled purple (purple ■), the P-2 molecule is labeled red (red ■), the P-3 molecule is labeled pink (pink ■), and the CO_2_ molecule is labeled ice-blue (iceblue ■); the box is in a Cartesian coordinate system, with a red arrow for the x-direction, a green arrow for the y-direction, and a blue arrow for the z-direction, and the figure illustrates the box on its y–z side. The model structure is set up in the VDW style, i.e., details affecting observation such as single- and double-bonds are hidden while keeping the molecular structure intact, and the molecule is shown as a molecular chain made of directly connected spherical atoms. To better determine the distribution of each molecule, the color was set to a transparent format to avoid the molecules at the back of the position being completely obscured.

According to the pattern of molecular distribution before and after polymer–CO_2_ equilibrium in Figure 5, it can be seen that since the initial state is an idealized design, only the distance distribution is considered to be homogeneous, and this process does not take into account the effect of the weak intermolecular interactions on the whole system, so the polymer and CO_2_ present an unrealistic homogeneous state in the initial state box. However, the molecules are not always in a uniformly distributed state within the system but are constantly moving and aggregating in the box until the system is stabilized. In addition, some P-n molecules can be observed to escape from the box in the figure, but the whole simulation is performed again under three-dimensional periodic boundary conditions, and the system follows the cyclic calculation rule. (Because the P-n molecular chain is relatively long, when the molecule moves to the edge of the box, part of the chain segment escapes from the top of the box; the part is not leaving the equilibrium calculation area, but re-entering the box from the bottom of the box; the overall molecular structure and the number of molecules will not change; the escaping part is just the image that the model automatically supplements to better express the law of molecular motion system.) Weak intermolecular interactions were examined throughout the kinetic simulations to characterize the aggregation behavior of the tackifier molecules throughout the system. Comparing the simulated initial and equilibrium structures, the equilibrium system calculated by MD shows that the molecular spatial distribution of the polymers in the equilibrium system changes under the action of van der Waals forces, the polymers gradually show aggregation among themselves, and the volume of the box collapses with the change in environment (temperature and pressure) to fill up the cavities generated by molecular motion inside the box, so that the box of the system is much smaller than that of the initial 2000 nm^3^, but the energy of the system is shown to be minimized. The side lengths (length, width, and height) of the box in each direction after the final system achieves equilibrium are measured during the simulation, and the final volume of the box is calculated as shown in Table 3.

When the system reaches the final calculated equilibrium, the CO_2_ molecules are stably distributed throughout the system, and the P-n molecules are mainly concentrated in the middle of the box. Figure 5 shows the distribution of CO_2_ on the central slice of the three equilibrium systems: some P-n molecules appear to be cross-aggregated throughout the movement due to the long chain segments, and the CO_2_ molecules are still wrapped up in the gaps between the chain segments of the P-n molecules in an independent form.

When the system reaches the final calculated equilibrium, the CO_2_ molecules are stably distributed throughout the system, and the P-n molecules are mainly concentrated in the middle of the box.

### 4.3. Examination of Polymer Molecular Distribution Laws

#### 4.3.1. Solubility of PVE in scCO_2_

**Figure 6:**
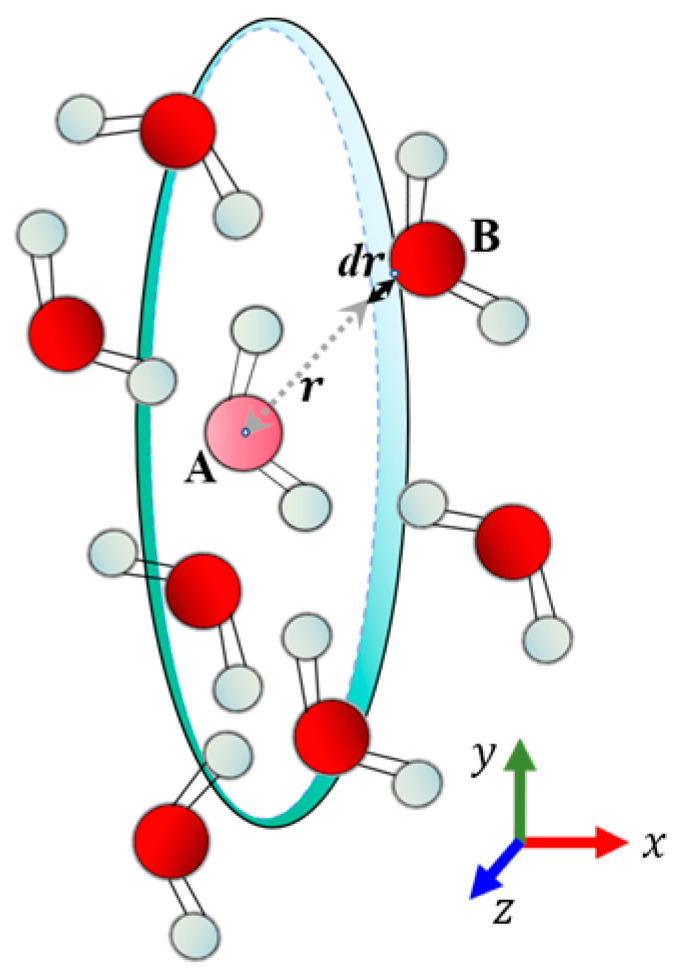
Radial distribution function schematic diagram.

The radial distribution function (RDF) investigates the density of B atoms within the shell at a distance r from the A atoms relative to the mean value of the density of B atoms in the whole box, g(r), and the principle of the RDF is shown schematically in Figure 6.

The equation defining g(r) between the type A example and the type B particle in Figure 6 is shown in Equation (7):

(7) $$g_{AB}\left(r\right)=\frac{\rho_{B}\left(r\right)}{\rho_{B_{Local}}}=\frac{1}{\rho_{B_{Local}}}\times \frac{1}{N_{A}}\sum_{i\in A}^{N_{A}}\sum_{i\in B}^{N_{B}}\frac{\sigma \left(r_{ij}-r\right)}{4\pi r^{2}}$$

where $\rho_{AB}$ and $\rho_{B_{Local}}$, respectively, denote the A-type particles around the distance r at the B-type particle density; all the *A* particles are at the center, within the radius of the r_max_ shell layer within the average density of *B* particles, and usually, the r_max_ value denotes half the box length.

**Figure 7:**
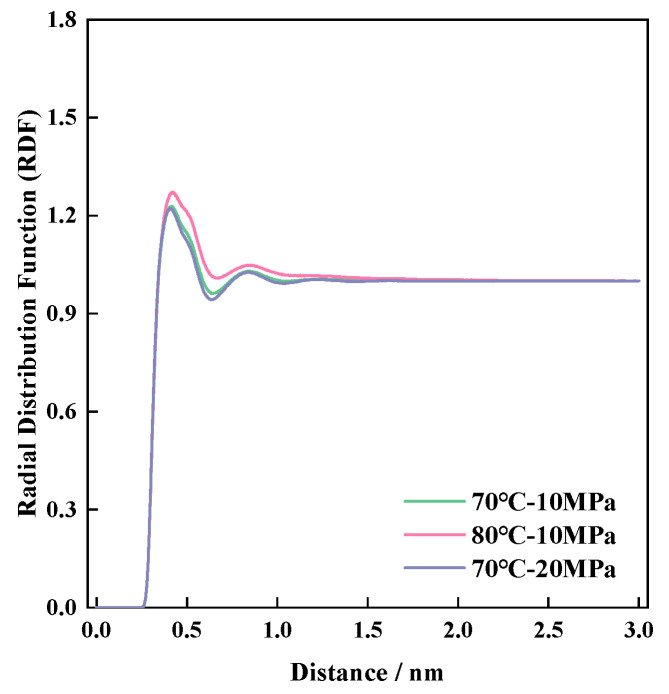
CO_2_-CO_2_ radial distribution function.

**Figure 8:**
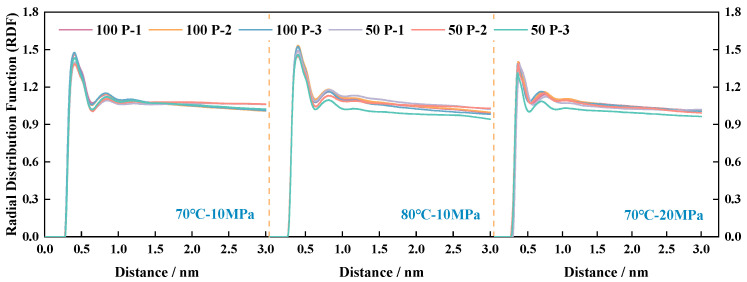
CO_2_ radial distribution function for three different polymers.

The g(r) of CO_2_-CO_2_ in the blank control group was first explored to see if it was affected by changes in the environmental factors of the system, see Figure 7. By comparing the RDF curves under different environmental conditions, it can be seen that the first peak in density at 0.408 nm occurs for CO_2_ under all three environmental conditions, and the *y*-axis value of the first peak represents the probability of CO_2_ presence in the nearest distance between CO_2_-CO_2_ under the three environmental conditions, which are 1.228, 1.267, and 1.222, respectively. The trend of the curve change shows that in addition to the first peak with the highest density, there is a wider short peak at 0.834 nm, which is the second closest distance between CO_2_-CO_2_, and the curve gradually flattens out to 1 with the increasing interatomic distance, according to which it can be confirmed that the probability normalization of the MD process is more effective. In the figure, we can also observe that the peak widths of the three curves are different: The molecular amplitude becomes larger at high CO_2_-CO_2_ temperatures and tends to leave the equilibrium position, while high pressure inhibits the relative molecular motion, which is manifested by the narrowing of the peaks and a decrease in peak height. The CO_2_-CO_2_ radial distributions were ranked for the pure CO_2_ system under different environmental conditions, from high to low: 80 °C-10 MPa >70 °C-10 MPa >70 °C-20 MPa. The radial distribution of the polymer–CO_2_ was next explored, and the distribution pattern of g(r) curves for the three polymers is plotted in Figure 8.

By comparing the fluctuation trend of RDF curves in Figure 7 and Figure 8, the change rule of g(r) curves of the polymer–CO_2_ system is consistent with that of the pure CO_2_ system, evidenced by the influence of the environment, but the intermolecular distance of the polymer–CO_2_ system is obviously higher than that of the pure CO_2_ system, and the peak position is also further away from that of the pure CO_2_ system before. This is mainly due to the large difference in the calculated volumes of the two molecules in the box and the presence of a large spatial site resistance, such that the distance between the CO_2_ and the polymer fraction is further than the distance between CO_2_ and CO_2_. The RDF curves of the three systems show a pattern wherein the greater the total number of atoms contained in the P-n molecule, the larger the g(r) peak, i.e., P-3 > P-2 > P-1. The larger the peak, the stronger the solubility of the polymer in CO_2_, i.e., the solubility of the polymer in CO_2_ is also in the following order: P-3 > P-2 > P-1. Comparing the number of P-n molecules in the same polymer–CO_2_ system with 50 or 100, respectively, the total number of molecules is progressively lower.

#### 4.3.2. System Density Change Rule

After experiencing the calculation of molecular distribution at MD equilibrium, there was a preliminary understanding of the different molecular motion degrees of freedom within the equilibrium system, but the RDF does not analyze the molecular distribution law within the system. The study of the molecular distribution laws within the system based on the molecular density distribution function involves the following formulas:

(8) $$\rho =\frac{N_{m}}{L^{3}}$$

where *N*_m_ is the number of particles, m (subscript) is the mass of the ion, and *L* is the length of the system box side.

**Figure 9:**
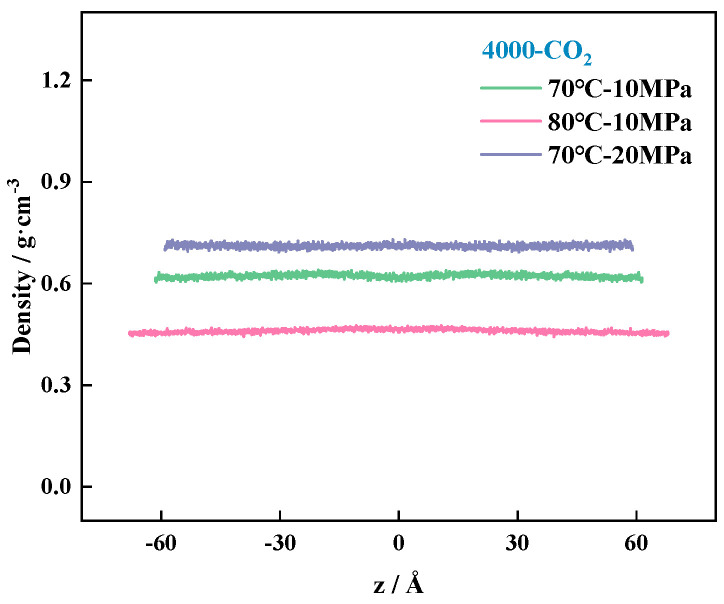
Molecular density distribution curves in pure CO_2_ systems under different environmental conditions.

**Figure 10:**
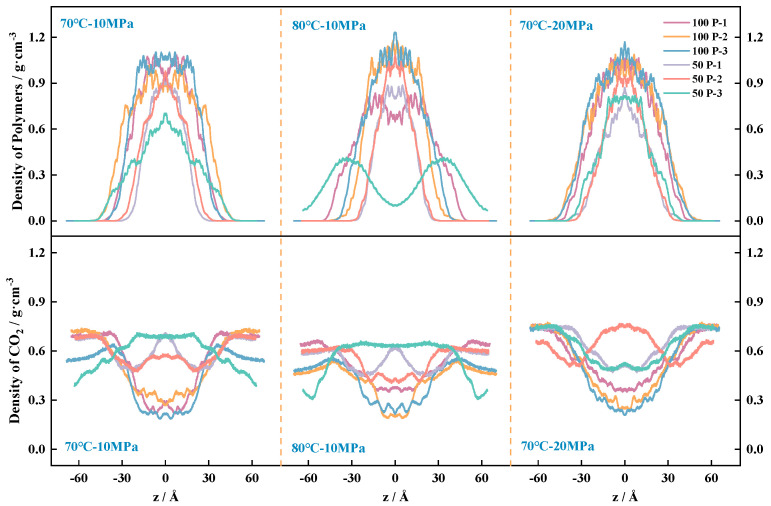
Molecular density distribution curves of polymer–CO_2_ system.

In Figure 9, we study the density distribution of the target molecules (CO_2_ and polymer) along the *z*-axis of the Cartesian coordinate system of the box, using the midpoint of the box as the coordinate origin, calculated taking into account that the system movement to the equilibrium state process between the molecules is not uniform distribution. To reduce the statistical error, the system along the *z*-axis is divided into 2000 cells, with the center of the system’s atoms as the reference point of the curve for the symmetrical optimization of the system, so as to obtain the density distribution curve of the pure CO_2_ system, as shown in Figure 9. The density curve of the two-component system is shown in Figure 10, and the unit of calculation of the density is g/cm^3^.

Figure 9 The smaller fluctuation range in the density curve indicates that the CO_2_ molecules are relatively uniformly stressed throughout the box, preventing large fluctuations due to changes in the system environment, and the density distribution behaves in a more even manner, so the density curve is smoother. Under high pressure, the curve fluctuates in a smaller range, which is affected by the pressure caused by the limitation of space compression transport; the temperature increases the expansion of the system; and the density is further reduced.

Figure 10 shows the molecular density distribution within the polymer–CO_2_ system. The curves in the upper half of the three figures represent the polymer density trend in the box under different environments, in which it can be observed that the density of the polymer is close to 0 at the ends of the box, while the density is higher near the center position, indicating that all P-n molecules are present in the middle of the box and form a large network of aggregated structures. The lower half of Figure 10 shows the CO_2_ density trend in the box under different environments. According to the pattern of the curves in the figure, after the introduction of the polymer, the distribution of the CO_2_ system is significantly different from that of the pure CO_2_ system, and the CO_2_ is not uniformly distributed throughout the box. Due to the good mutual solubility between the polymer and CO_2_ [35], part of the CO_2_ exists in the voids of the polymer aggregation network structure in the center of the box; the CO_2_ in the system tends to be more oriented towards the vacuum in comparison with the polymer, indicating that the interaction force between the P-n molecules is higher than the degree of interaction between the polymer and CO_2_; and it can form a stabilized cage structure, thus limiting the CO_2_ transport rate. The viscosity enhancement of the system is finally realized. The distribution pattern obtained in Figure 10 is consistent with the aggregated state of molecular clusters embodied in Figure 4.

When different viscosity enhancers are introduced, changes in the system equilibrium cause changes in the density profile, and the density fluctuation curves of the CO_2_ and polymers within the same system have opposite patterns, but the sum of the values of the curves per unit distance tends to be the same. For the same number of molecules, increasing the pressure results in a more similar density distribution curve width, at which point the proportion of VAc on the molecular chain has less effect on the CO_2_ density distribution. Lowering the temperature decreases the intermolecular thermal motion, which is shown in the curve as a broadening in the curve when the role of the pro-CO_2_ group increases significantly and the density rises. When increasing the number of polymers, the density of P-n molecules is lower under the same environmental conditions; in terms of the width of the density distribution, the higher the amount of VAc bound on the chain of P-n molecules, the more stable the density distribution of polymers in the system is [21]. Comparison shows that the temperature increase has the most obvious effect on the density distribution of polymer P-3, which is because the degree of stretching in the molecular chain itself is more obviously affected by the pro-CO_2_ C=O: the temperature increases, the molecular chain stretches, the P-3 molecular chain is the longest, there is less space between the molecules, and the density of CO_2_ that can be transported in it decreases. When the molecular chain is stretched to a certain extent, the movable space in the system is completely occupied, at which time the CO_2_ molecules in the system are no longer significantly displaced.

#### 4.3.3. Laws of Change of CO_2_ and Surface Area Before and After Equilibration

The contact area determines the degree of fusion of the two molecules in the system. To further study the contact between aggregates and CO_2_, the total contact accessible surface area (Solvent Accessible Surface Area (SASA)) of the system solvent CO_2_ to the solute polymers was calculated as shown in Equation (9). The calculation uses the CO_2_ molecule as the reference origin and 0.14 nm as the radius, where *m_ace_*(*i*) is the sum of the number of atoms on the P-n molecule that are not obscured by neighboring molecules; *r_i_* is the radius of the trajectory of the P-n molecule.

(9) $$A=4\pi \sum_{i}r_{i}^{2}\frac{m_{ace}\left(i\right)}{m}$$

**Figure 11:**
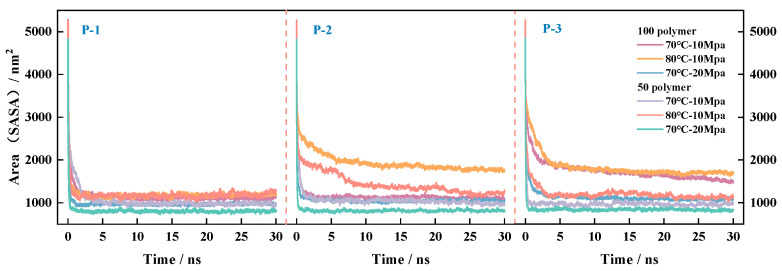
Surface area curves of CO_2_ and polymer under different temperature and pressure environments.

The convergence curve of the total contact area is based on the calculations shown in Figure 11. The order of energy magnitude of the system before equilibrium remains essentially the same, independent of the system environment. According to the fluctuation trend in the curves, it can be seen that the total area of polymer–CO_2_ contact is larger initially, which is due to the initial structure of the P-n molecules with CO_2_ molecules in a disordered distribution state; the total area of contact between the molecules is larger; the MD simulation reaches equilibrium under different environmental conditions; and the systems all converge to a stable surface area (nm^2^) rapidly, in which the accessible surface area of P-1 and CO_2_ is affected by the least environmental influence. Under the effect of molecular thermal motion, the transport of small solvent molecules (CO_2_) in the system voids accelerates with increasing temperature, which is not conducive to the maintenance of system viscosity, although it increases the system SASA. Elevating the pressure will have an inhibitory effect on the molecular movement while decreasing the SASA of the system, at which time the contact area size per unit volume is more stable, which in turn helps to increase the viscosity of the system. Under the same environmental conditions, the contact area of the same polymer increases with the number of molecules. Under different environmental conditions, an increase in the number of atoms in the molecular structure (the higher the proportion of VAc) increases the SASA of solvent CO_2_, indicating that the C=O pro-CO_2_ group promotes the degree of stretching of the molecular chain segments, which is conducive to increasing the polymer’s contact area with CO_2_. Combined with the phenomenon of increasing viscosity with increasing molecular weight that appeared in the previous work, it shows that for the same viscosity enhancer, the SASA between CO_2_ and P-n molecules is positively correlated with the viscosity of the system, which explains why all three polymers can achieve significant viscosity enhancement.

### 4.4. Examination of Functional Groups and Atoms of Polymer Molecules and CO_2_ Interaction Law

#### 4.4.1. Analysis of Fluctuations in the Radius of Gyration

**Figure 12:**
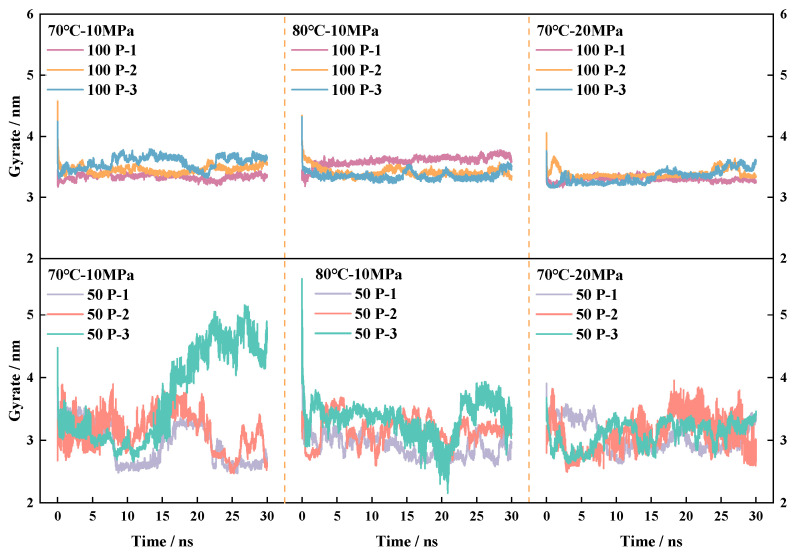
Cyclotron radius changes in three polymer molecules under different environments.

In the sections discussing molecular distribution, density, and CO_2_-accessible surface area, reference is made to the degree of molecular chain stretching, i.e., the ability of the molecule to bend and stretch, a molecular kinematic behavior that is primarily caused by changes in molecular radius gyration. The radius of gyration (ROG) is a commonly used metric for evaluating the weighted average radius of mass and the closeness of molecules during the MD process and was investigated for the gyration change curves of P-n molecules under different system conditions, respectively, as shown in Figure 12, with the calculations relying on Equations (10) and (11).

(10) $$R_{g}=\sqrt{\sum_{i}m_{i}{\left({\hat{R}}_{i}-{\hat{R}}_{c}\right)}^{2}/\sum_{i}m_{i}}$$

(11) $$R_{g}\left(x\right)=\sqrt{\sum_{i}m_{i}\left(R_{i}{\left(y\right)}^{2}-R_{i}{\left(z\right)}^{2}\right)/\sum_{i}m_{i}}$$

where *m_i_*, *R_i_*, *R_g_*, and (*y*/*z*) are the proton mass, the coordinates of the *i* atom, the center of mass of the system, and the distance between the atom and the center of mass of the system in the *y*/*z* direction, respectively.

Figure 12 shows that under the action of molecular thermal motion, the radius of gyration of the molecules in the system is dynamic, basically having reached a relatively stable degree of gyration at 25~30 ns. As can be seen from the magnitude of the radius of gyration fluctuations in the graph at 70 °C, increasing either the temperature or the pressure can inhibit the degree of the radius of gyration fluctuations of the P-n molecules in the system to a certain extent. The larger the percentage of polymer density per unit volume, the smaller and less flexible the molecule’s cyclotron space (the space in the box where the polymer molecular chains are free to slew and coil) is, so P-3, which has the largest total number of atoms and the longest molecular chain, exhibits more pronounced cyclotron limitation in the 100-polymer–CO_2_ box. Among the three polymers, P-2 has the most stable fluctuation in radius of gyration, which is because the P-2 molecular structure is completely symmetric and the forces on both sides of the central atom are more balanced, so the fluctuation range is less shifted. The smaller the radius of gyration, the denser the molecular arrangement in the system, the lower the relative motion of the polymer, and the lower the opportunity for CO_2_ to enter the pore space of the polymer network, thus reducing the mutual solubility between the polymer and CO_2_, which is not conducive to the viscosity enhancement of the system. The radius of gyration of the polymer in the 50-polymer–CO_2_ box is significantly larger than that in the 100-polymer–CO_2_ box, indicating that the larger the radius of gyration of the molecules in the same system, the more the molecular chain is stretched out, the more the molecules occupy the space within the system box, and the more the molecular chain builds up a sparser spatial network structure, which is conducive to the transportation of CO_2_. At 70 °C-10 MPa, the 50 P-3 system showed a significant increase. The fluctuation was larger than in the other systems, which is caused by the elevated values of radius of gyration and increased molecular chain coiling in 15–30 ns. When 100 polymers are introduced, the degree of intermolecular aggregation is higher, limiting to some extent the possibility of CO_2_ entering the pores of the polymer network, and the increase in the degree of mixing between the two molecules is not the same as the increase in the number of polymers.

It is worth noting that the central problem of the molecular dynamics simulation algorithm is to ensure that the calculation of the force, regardless of the movement of the particles in the system, does not affect the system energy conservation. Combined with the radial distribution curve, the density data, the CO_2_-accessible surface area, and the radius of gyration curves, it can be seen that the stability of the system is good.

#### 4.4.2. Calculation of the Minimum Intermolecular Contact Distance and the Number of Pairs of Contacting Atoms

The minimum intermolecular contact distance and the number of pairs of contacting atoms were calculated to more intuitively characterize the actual viscosity enhancement and solubilization effects of the P-n-CO_2_ system. When the calculated equilibrium is reached, the molecular displacement in the system decreases and the number of molecular contacts basically tends to a constant value, so the last 5 ns of the finished product simulation was used as the object of this study and the dynamic mean value of the minimum distance (Mindistance) of the H-C between the two components (polymer–CO_2_) was calculated, respectively [36], and the results of the calculations are shown in Table 4; additionally, the number of pairs of atomic contacts are shown in Table 5. According to Table 4, it can be seen that, under the same environmental conditions, the greater the number of molecules in the same system, the smaller the intermolecular distance. The molecular structure is different: the stronger the symmetry of the molecular structure, the smaller the intermolecular distance, which is consistent with the change rule of the radius of gyration in Section 4.4.1. Due to the molecular symmetry of the molecules, the strength of the vibration amplitude of the molecules is lower, resulting in the P-2 in the system of the contact with the CO_2_ being slightly lower than that of the other two viscosities. The change in the radius of gyration in Section 4.4.1 is the same.

**Figure 13:**
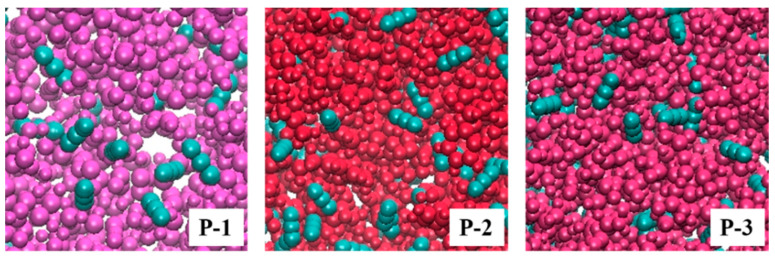
Box slices of CO_2_ molecule distribution in different polymer systems.

After calculating the equilibrium, the molecular spacings are all significantly larger than the C-H bond lengths of 1.09. Both CO_2_ molecules and P-n molecules in the system remain structurally intact, and there are no bond breaks or bridges between molecules in Figure 13. Some P-n molecules appear to be cross-aggregated throughout the movement due to the long chain segments, and the CO_2_ molecules are still wrapped up in the gaps between the chain segments of the P-n molecules in an independent form. This indicates that there is no chemical reaction between polymer–polymer and polymer–CO_2_, but rather intermolecular forces maintain the equilibrium of the system, i.e., the stable coexistence of polymer and carbon dioxide can be realized under the conditions of 70 °C-10 MPa, 80 °C-10 MPa, and 70 °C-20 MPa.

By comparing the number of pairs of atoms in contact in the system at equilibrium, it can be seen that the higher the total number of atoms contained in the P-n molecule, the higher the number of atoms in contact, since the three polymers differ only in the number of VAc fragments introduced into the main chain. It is shown that the introduction of VAc fragments can improve the contact efficiency of the polymer with CO_2_, which is consistent with the conclusions obtained from the analysis of density and radial distribution, indicating that the introduction of VAc can promote the affinity between the two components of the system, i.e., to promote the occurrence of the polymer’s solubilization behavior in CO_2_, which will, in turn, improve the overall viscosity increase in the system.

## 5. Conclusions

To further analyze the viscosity enhancement mechanism of P-n series viscosity enhancers on CO_2_, based on previous experiments, P-n series polymer molecules with a linear symmetrical structure were designed by adjusting the ratio of the number of substances of each functional group of the polymer molecules, and molecular dynamics simulation of P-n molecules was carried out, which revealed the viscosity enhancement mechanism of the polymer P-n in the CO_2_ system, as follows:

- The viscosities of the P-n-CO_2_ systems, in descending order, are Ƞ_100-P-3_, Ƞ_100-P-2_, Ƞ_100-P-1_, Ƞ_50-P-3_, Ƞ_50-P-2_, and Ƞ_100-P-1_.
- All of the above systems achieved effective viscosity enhancement, and the degree of viscosity enhancement of P-n molecules was positively correlated with the contact area of CO_2_ and the number of P-n molecules.
- The molecules within the equilibrium system did not occur between the phenomena of bond breaking, bridging, etc.; that is, there was no chemical reaction.
- Multi-scale analysis of microscopic interaction patterns between P-n molecular structures and CO_2_ molecules: the molecular weight was positively correlated with the molecular amplitude, radial distribution peak, molecular radius of gyration, and effective contact area.
- For the molecules containing the total number of atoms, the molecular density distribution of the system tends to be more stabilized, allowing for a greater number of atom contact pairs.
- The minimum space between P-n molecules and CO_2_ molecules in the system model was calculated to be in the range of 1.699–1.736 Å. The introduction of VAc can promote the dissolution of polymers in CO_2_.

## Figures and Tables

**Table 1:** Atomic composition of the three polymers.

Name of Polymer	Component Mole Ratio St/MA/VAc	Atomic Number Ratio C/H/O	Total Number of Atoms
P-1	1:4:1	56:60:28	144
P-2	1:4:2	64:78:44	168
P-3 ^1^	1:4:2.5	68:78:44	190

^1^ For ease of analysis, this paper writes PVE (1:4:2.5) as P-3.

**Table 2:** Change in dihedral angle before and after mechanical optimization of polymer functional groups.

Two-Sided Angle	Molecular Conformation	Initial Drawing of Structures/°	P-1	P-2	P-3
C-Phenyl	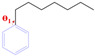	65.61	109.78	63.87	120.22
C- Carboxy	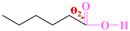	128.45	94.26	100.17	126.26
C-Ester	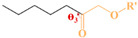	125.24	108.29	109.31	114.67

**Table 3:** Change in box volume after MD balance/nm^3^.

Environmental Settings	50 Polymers-CO_2_			100 Polymers-CO_2_			CO_2_
	P-1	P-2	P-3	P-1	P-2	P-3	
70 °C-10 MPa	483.68	490.30	499.38	554.37	553.18	640.51	464.78
80 °C-10 MPa	542.43	541.10	520.02	563.91	689.79	686.48	631.29
70 °C-15 MPa	455.40	462.68	472.00	521.06	543.80	563.15	411.76

**Table 4:** Minimum distance/angstrom (Å).

Theoretical Viscosity	100			50		
Environmental Settings	P-1	P-2	P-3	P-1	P-2	P-3
70 °C-10 MPa	1.706	1.670	1.709	1.736	1.730	1.735
80 °C-10 MPa	1.704	1.708	1.709	1.735	1.736	1.736
70 °C-15 MPa	1.703	1.699	1.703	1.727	1.731	1.730

**Table 5:** Atomic contact logarithm.

Theoretical Viscosity	100			50		
Environmental Settings	P-1	P-2	P-3	P-1	P-2	P-3
70 °C-10 MPa	202,189	217,560	225,079	105,411	121,834	134,987
80 °C-10 MPa	208,187	208,932	213,365	106,011	116,343	129,021
70 °C-15 MPa	207,601	246,558	248,779	127,586	137,326	143,336

## Data Availability

Data are contained within this article.
